# Assessment of compliance with good review practices by medicine assessors within the Zambia medicines regulatory authority

**DOI:** 10.3389/fmed.2025.1706139

**Published:** 2025-11-11

**Authors:** Constance Sakala Chisha, Stephanie Leigh, Makomani Siyanga, Neil McAuslane, Stuart Walker

**Affiliations:** 1 Department of Pharmacy and Pharmacology, University of the Witwatersrand, Johannesburg, South Africa; 2 Zambia Medicines Regulatory Authority, Lusaka, Zambia; 3 WITS Pharmacy Regulatory Science Hub, Department of Pharmacy and Pharmacology, School of Therapeutic Sciences, Faculty of Health Sciences, University of the Witwatersrand, Johannesburg, South Africa; 4 Centre for Innovation in Regulatory Science, London, United Kingdom

**Keywords:** good review practices, regulatory review, optimising efficiencies in regulatory agencies (OpERA), WHO listed authority, maturity level

## Abstract

**Introduction:**

The implementation of Good Review Practices (GRevPs) ensures the timely, high-quality review and enhanced availability to safe, quality, and efficacious medicines. It is important, therefore, that all aspects of GRevPs are continuously updated and monitored to promote improvement of the review process.

**Methods:**

This study aimed to assess the implementation of the GRevPs in the Zambia Medicines Regulatory Authority (ZAMRA) to identify opportunities for improvement. The GRevP Questionnaire developed by the Centre for Innovation in Regulatory Science (CIRS), an established, structured and multi-dimensional questionnaire was completed by the scientific reviewers of the ZAMRA.

**Results:**

Twelve of the 16 assessors took part in the study of whom 5 (42%) reported that GRevPs have been implemented and fully adopted across the agency. The study participants provided details indicating the reasons why they believe quality measures had been implemented within ZAMRA. The three most common reasons were to be more efficient, to improve process predictability and increase transparency. However, most of the respondents believed that the current GRevPs framework could be improved. The study noted that training on how GRevPs are to be used and incorporated into daily work was deemed valuable. All the participants reported that ZAMRA has a consistent method for documenting those practices that need to be improved by GRevPs. A mechanism to facilitate updating GrevP at ZAMRA is currently in place, however, it requires enhancement. In general, the importance of GRevPs was well understood by the assessors, however the study showed that target timelines were not well followed at both the department and agency levels.

**Discussion:**

This study has successfully determined the GRevPs used in the review process and their implementation by ZAMRA. It has provided a reference point from which the ZAMRA can work towards improvement as a means to enhance regulatory performance. The findings of this study will form the basis of development for the NRA as it strives to achieve the WHO Maturity Level 3 status.

## 1 Introduction

Access to effective, safe and quality-assured medicines is a global public health priority (1, 2). National Regulatory Authorities (NRAs) are essential in managing medicines in protecting public health (3, 4). These NRAs are responsible for enforcing regulations and issuing guidelines for various aspects of pharmaceutical development, manufacturing and distribution of medicines (29). Well-functioning NRAs are key in strengthening the healthcare system (2). However, many NRAs, particularly in low- and middle-income countries (LMICs), have limited capacity to assess and approve health products due to inadequate human resources, weak regulatory systems, inadequate surveillance infrastructure, automation systems and incoherent policy frameworks (5, 6, 30). To address these issues and promote global access to quality medicines, various modalities have been developed to improve regulatory capacity and harmonise standards (4). These include adoption of international standards and practices such as the World Health Organisation (WHO’s) Good Regulatory Practices and Global Benchmarking Tool (7), fostering collaboration through Regional Economic Communities (RECs), Good Reliance Practices, and quality management systems (5).

Strengthened regulatory systems of medicines are critical in ensuring access to high-quality, safe, and efficacious medicines (8). This can be achieved by adopting comprehensive frameworks, harmonising technical requirements, building capacity, and addressing restraint resources (9). The technical standards for submissions have been harmonised via the International Council for Harmonisation of Technical Requirements for Pharmaceuticals for Human Use (10). Quality attributes require the establishment of solid quality management tools and the maintenance of an appropriate regulatory system (13).

Good Review Practices (GRevPs) are defined as ‘documented evidence of any aspect of the process, format, content, and management of the regulatory review’ and they form an integral part of the review process (11). These practices were developed to achieve timeliness, predictability, consistency, transparency, clarity, efficiency and high quality in the content and management of the reviews (12), in so doing standardising the review process (11). All this is achieved through the development of the guidelines, standard operating procedures, evaluation templates, staff training and orientation packages, and having quality decision-making frameworks (13). Others include established target times, parallel dossier assessment, and continuous improvement processes (11). These are considered as the building blocks of the quality review process (13). A good review is balanced, considers context, is evidence based, identifies signal, investigates and solves problem, makes linkages, utilises critical analyses, is thorough, well-documented and well managed (11).

The GRevP guideline was first developed by the expert working group that was convened by the Asia-Pacific Economic Cooperation (APEC) Regulatory Harmonisation Steering Committee with WHO in 2013 (11).

**Table 1:** 

**Ten key principles of a good review** *Balanced* A good review is objective and unbiased. *Considers context* A good review considers the data and the conclusions of the applicant in the context of the proposed conditions of use and storage, and may include perspectives from patients, health-care professionals and other RAs’ analyses and decisions. *Evidence-based* A good review is evidence-based and reflects both the scientific and regulatory state of the art. It integrates legislative, regulatory and policy frameworks with emerging science. *Identifies signals* A good review comprehensively highlights potential areas of concern identified by the applicant and the reviewers. *Investigates and solves problems* A good review provides both the applicants and the reviewers’ in-depth analyses and findings of key scientific data and uses problem-solving, regulatory flexibility, risk-based analyses and synthesis skills to devise and recommend solutions and alternatives where needed. *Makes linkages* A good review provides integrated analysis across all aspects of the application: preclinical,	nonclinical, clinical, chemistry/biocompatibility, manufacturing and risk management plan. It includes timely communication and consultation with applicants, internal stakeholders and, as needed, with external stakeholders who have expertise relevant to the various aspects of the application. *Utilises critical analyses* A good review assesses the scientific integrity, relevance and completeness of the data and proposed labelling, as well as the interpretation thereof, presented in the application. *Thorough* A good review reflects adequate follow through of all the issues by the reviewers. *Well-documented* A good review provides a well-written and thorough report of the evidence-based findings and conclusions provided by the applicant in the dossier, and the reviewers’ assessment of the conclusions and rationale for reaching a decision. It contains clear, succinct recommendations that can stand up to scrutiny by all the parties involved and could be leveraged by others. *Well-managed* A good review applies project and quality management processes, including clearly defined steps with specific activities and targets.

Adopted from World Health Organisation (11).

This was the first set of guidelines that was developed to address the gap identified in the review process (11). Good Review Practices (GRevPs) have been widely promoted to improve the efficiency and quality of regulatory review processes (11). These benefits are particularly critical for low- and middle-income countries, where resource limitations may hinder effective regulation. However, for GRevPs to remain effective, they must be routinely evaluated and updated to reflect evolving scientific and regulatory standards (13).

Several studies have assessed GRevPs implementation across several countries in the East African Community (EAC) Region (31), Western (12) and Southern Africa, offering valuable insights into common strengths and gaps in the review process.

The Zambia Medicines Regulatory Authority (ZAMRA) is the statutory body mandated by law to regulate medicines. The Marketing Authorisation Department is responsible for the registration of medicines, and three review models are used mainly verification, abridged and full review (14). The review process involves validation, scientific assessment and approval or rejection of the application (14). The Authority receives an average of 40 human medicine applications monthly most of which are generics as compared to New Chemical entities. The Authority has set target timelines based on the type of application and product (ZAMRA Service Delivery Charter 2024). The principles of the ZAMRA Good Review practices include fair, objectivity, timeliness and transparency. This is demonstrated through the use of standard operating procedures, assessment templates and reports (14). A study conducted by Sithole et al. (15) and more recently by Chisha et al. (14) demonstrated that the Authority implements certain elements of GRevPS in its review process. The retrospective study conducted by Chisha et al. (14) was undertaken to assess the regulatory review process of ZAMRA, focusing on products approved between 2020 and 2023. The aim was to determine achievement of key milestones, evaluate target timelines, and determine the extent to which good review and quality decision-making practices were implemented. Data was systematically collected using the validated Optimising Efficiencies in Regulatory Agencies (OpERA) questionnaire and accompanying data collection template developed by the Centre for Innovation in Regulatory Science (CIRS). Findings indicated that while foundational GRevP were in place, areas for further improvement included the establishment of defined target timelines for each milestone in the review process, the introduction of structured training in quality decision-making processes, and the enhancement of staff development. Additional improvements suggested included the implementation of external peer review for New Active Substance (NAS) applications, formal training for applicants in dossier compilation to improve submission quality, and the development of a structured framework for engaging and monitoring external assessors. Lastly, ZAMRA would benefit from a comprehensive monitoring and evaluation system to track review efficiency, training outcomes, and regulatory performance across all operational areas (14). These findings inform and aim to complement the current study, which is focused specifically on reviewing ZAMRA’s internal processes and internal implementation of GRevPs.

Few studies have focused specifically on reviewing ZAMRA’s internal processes and while Zambia is part of the regional efforts, internal implementation of GRevPs has not been empirically evaluated, representing a critical knowledge gap. As the Authority strives to attain the WHO Maturity level 3 status in its regulatory functions, the Authority must be able to demonstrate continuous improvement in the review process by identifying their gaps and improving on them.

This study, therefore, aimed to evaluate the good review practices that are implemented in the review process and identifying the opportunities for improvement.

## 2 Methodology

### 2.1 Ethical approval

The study was granted ethical approval by the Human Research Ethics Committee (Medical) of the University of Witwatersrand, Johannesburg, South Africa (Waiver number: R14/49 Chisha).

Permission was granted by the ZAMRA for the collection of data and for its subsequent publication.

### 2.2 Data collection tool

The structured Good Review Practices questionnaire, developed by the Centre for Innovation in Regulatory Science (CIRS), was used in the study. The questionnaire characterises how Good Review Practices are developed, implemented, used, and refined within the ZAMRA. The tool consists of 17 different questions intended to establish the assessors’ level of knowledge, attitude, and practices regarding GRevPs. The questions are designed to ascertain whether the participants understand the development, adoption, and implementation of GRevPs.

The questionnaire was electronically distributed to the reviewers within the Marketing Authorisation Department and those who consented completed the questionnaire and those who did not consent to the study did not return the questionnaire.

The data collected was analysed using Microsoft Excel.

The objectives of the study were to:

- Identify the current reviewers’ perspective of ZAMRA in the use of GRevPs.
- Provide a baseline on the reviewers’ knowledge, attitudes, and practices, as well as identify barriers and areas for improvement.
- Explore the processes and procedures currently in place that relate to GRevPs.
- Determine how these procedures relate to the continuous process improvement within ZAMRA.

## 3 Results

The study results have been presented in three parts, including an assessment of knowledge of GRevP, practice, and attitude.

**Part I—Knowledge**: This describes how GRevPs are being developed within ZAMRA, whether GRevPs improve the performance of the Authority as well as the Marketing Authorisation Department, and how important GRevPs are to the department and the Authority.

**Part II—Practice**: This describes the development and adoption of GRevP, its implementation and maintenance, and the processes of informing staff, as well as testing, and improving GRevP.

**Part III—Attitude**: This includes assessment of satisfaction with the framework and process for the development of GRevP, how well staff rate GRevP development in terms of achieving ZAMRA goals and their support of review activities, what aspects still require GRevP and what could be done to improve implementation, and how well GRevPs are followed at both the department and ZAMRA level.

### 3.1 Part I—knowledge

The study was a census of all 16 reviewers employed within the Marketing Authorisation Department at the time of the study. Twelve out of sixteen (75%) assessors from the Marketing Authorisation Department completed the GRevP-specific questionnaire for the assessment of the good review practices by the Authority. Reviewers or assessors are trained professionals with backgrounds in pharmacy or medical scientists, most having attained WHO Level II or III competency. ZAMRA continues to enhance assessor capacity through ongoing training and mentorship programmes.

When asked to what extent they felt GRevPs are in development at ZAMRA, a total of five assessors indicated that GRevPs have been developed and fully adopted in the department’s daily practice. Three other assessors indicated that the best practices for key areas were in the process of being developed and an additional three assessors indicated that the good review practices have been developed but not yet adopted in the department’s daily practice. Additionally, two assessors indicated that GRevPs have been developed but have not yet been adopted in the department’s daily practice. It was observed that one assessor provided two answers. The variation in the responses points to a lack of understanding of the aspect of good review practices by majority of the assessors.

Respondents provided details indicating the reasons for introducing quality measures in ZAMRA. The three most common reasons were to increase transparency, be more efficient and improve process predictability.

### 3.2 Part II—practice

A total of 11 participants (90%) responded to the question “*In your view, how has ZAMRA adopted GRevP*?.” A total of 8 assessors (67%) responded that ZAMRA has formally adopted GRevP through the use of standard operating procedures, training, and compliance monitoring, while 3 (25%) responded that ZAMRA informally adopted GRevP through the availability of procedures but with little or no compliance monitoring. One respondent (8%) did not respond.

**Figure 1:**
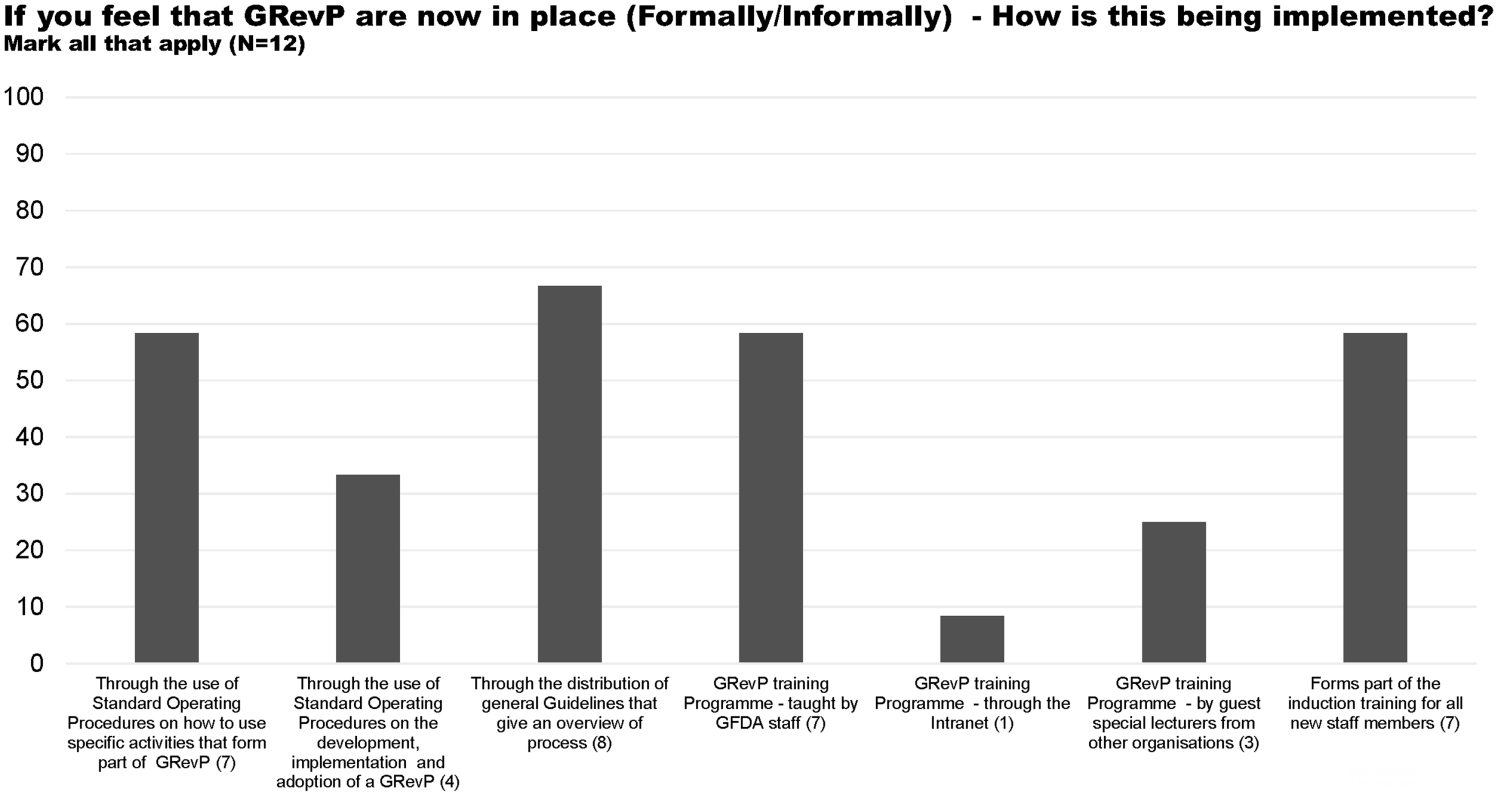
How GRevP is being implemented.

All of the 12 study participants believed that GRevPs are being implemented by the Authority either formally or informally (Figure 1). This is being done through the distribution of general guidelines that give an overview of the process, through the use of standard operating procedures on how to use specific activities that form part of the GRevP, as well as the GRevP training programme taught by ZAMRA staff and that it forms part of the induction training for all new staff members.

Three respondents indicated that GRevP is implemented through the GRevP training programme by guest special lecturers from other organisations, and one believed that it is implemented through GRevP training through the intranet (Figure 1).

**Figure 2:**
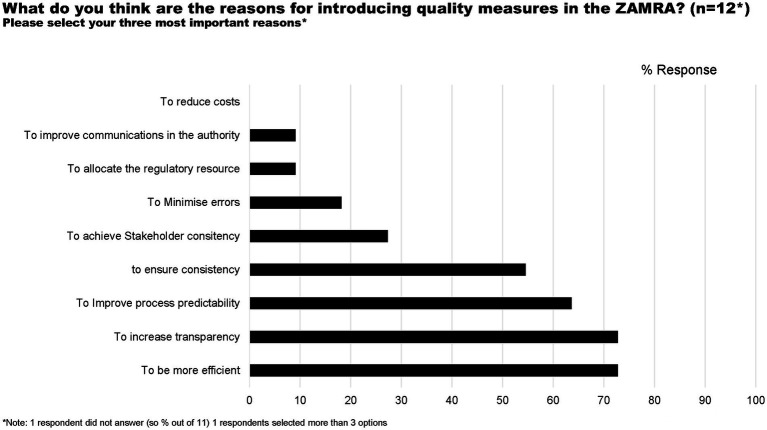
Reasons for introducing quality measures in ZAMRA.

The majority of study participants indicated that quality measures are introduced to the Authority to increase transparency (72%), efficiency (72%), and improved predictability (62%; Figure 2).

### 3.3 Part III—attitude

The study participants were then asked several “attitude related questions” as a means to establish an understanding of their satisfaction with the framework and process for the implementation of GRevPs. In response to the question of whether or not the assessors were satisfied with the existing GRevPs framework applied at ZAMRA, a total of 9 out of 11 (82%) respondents indicated that the framework could be improved, while 1 respondent (9%) indicated that they are satisfied. One respondent indicated that they were not satisfied with the framework and the implementation process of GRevPs.

The respondents indicated that the GRevP framework needed improvement to enhance consistency and transparency in the review process. Among those who suggested the framework could be enhanced, approximately 8 out of 11 (72%) believed that the GRevP system is still evolving within ZAMRA and is not yet complete. Five respondents (48%) felt that additional training is required to understand and learn how to use and incorporate GRevP into their daily work. Two (19%) responded that the benefits of implementing the GRevP system are not yet apparent to the ZAMRA management.

A total 7 out of 12 respondents (58.3%) indicated that GRevP has been developed based on the best practices identified through the collective experience of ZAMRA and the review teams, while 3 respondents (25%) indicated that the GRevP are based largely on the best practices identified from the activities of other agencies which are then adopted by ZAMRA; 1 respondent (8.3%) indicated that the GRevP have been developed by the review management team, and it reflects their view of the practices that should be adhered to. All the participants indicated that there are still best practices that need to be implemented including feedback from companies, feedback from staff/assessment team, standard operating procedures, quality department, internal audit, feedback from patients, ability to track review process, quality policy, and target timelines.

**Figure 3:**
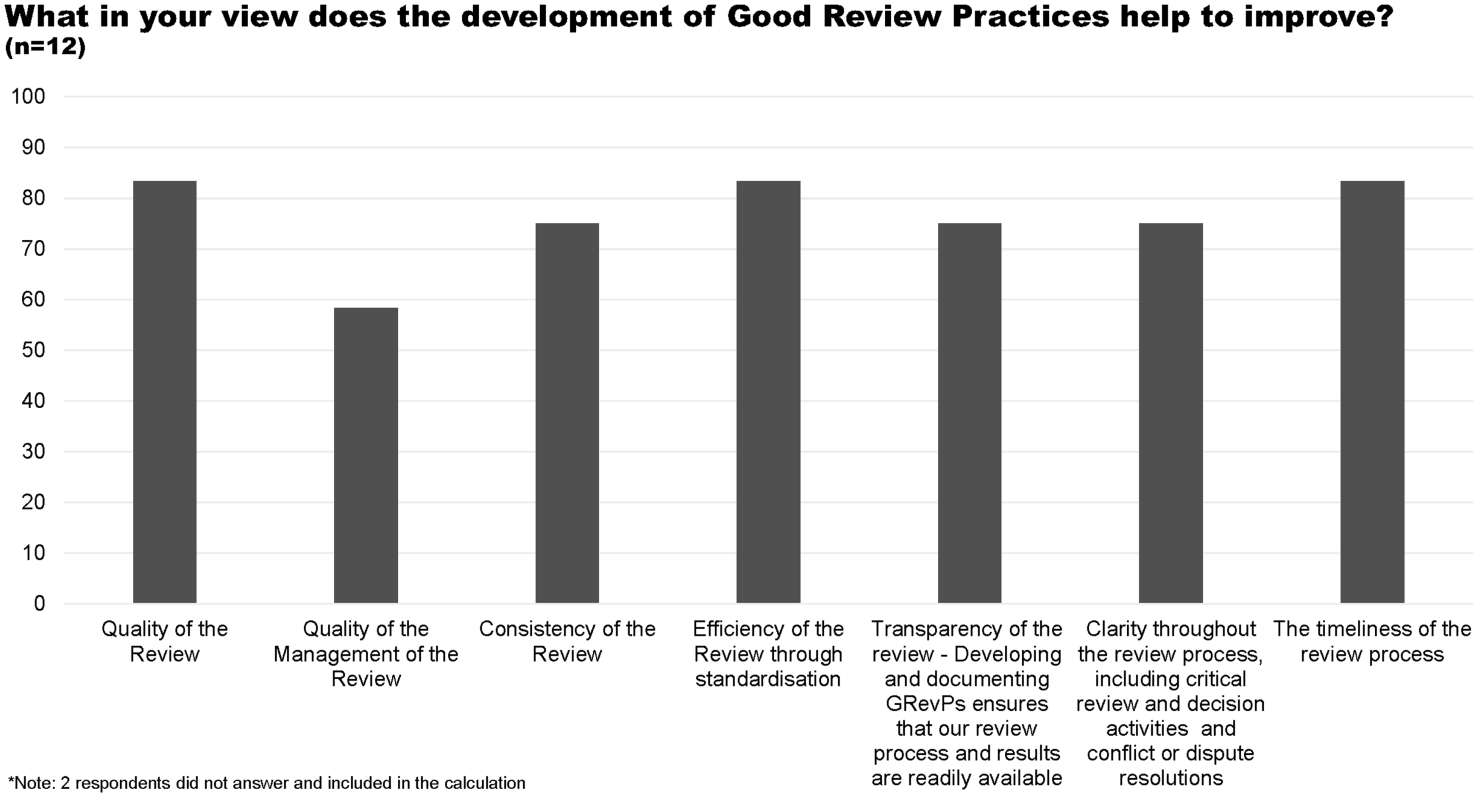
Elements of the review improved through GRevP.

Most of the respondents believe that the development of GRevP helps to improve the quality and timelines of the review, efficiency of the review through standardisation, consistency of the review, transparency, and clarity throughout the review (Figure 3).

**Figure 4:**
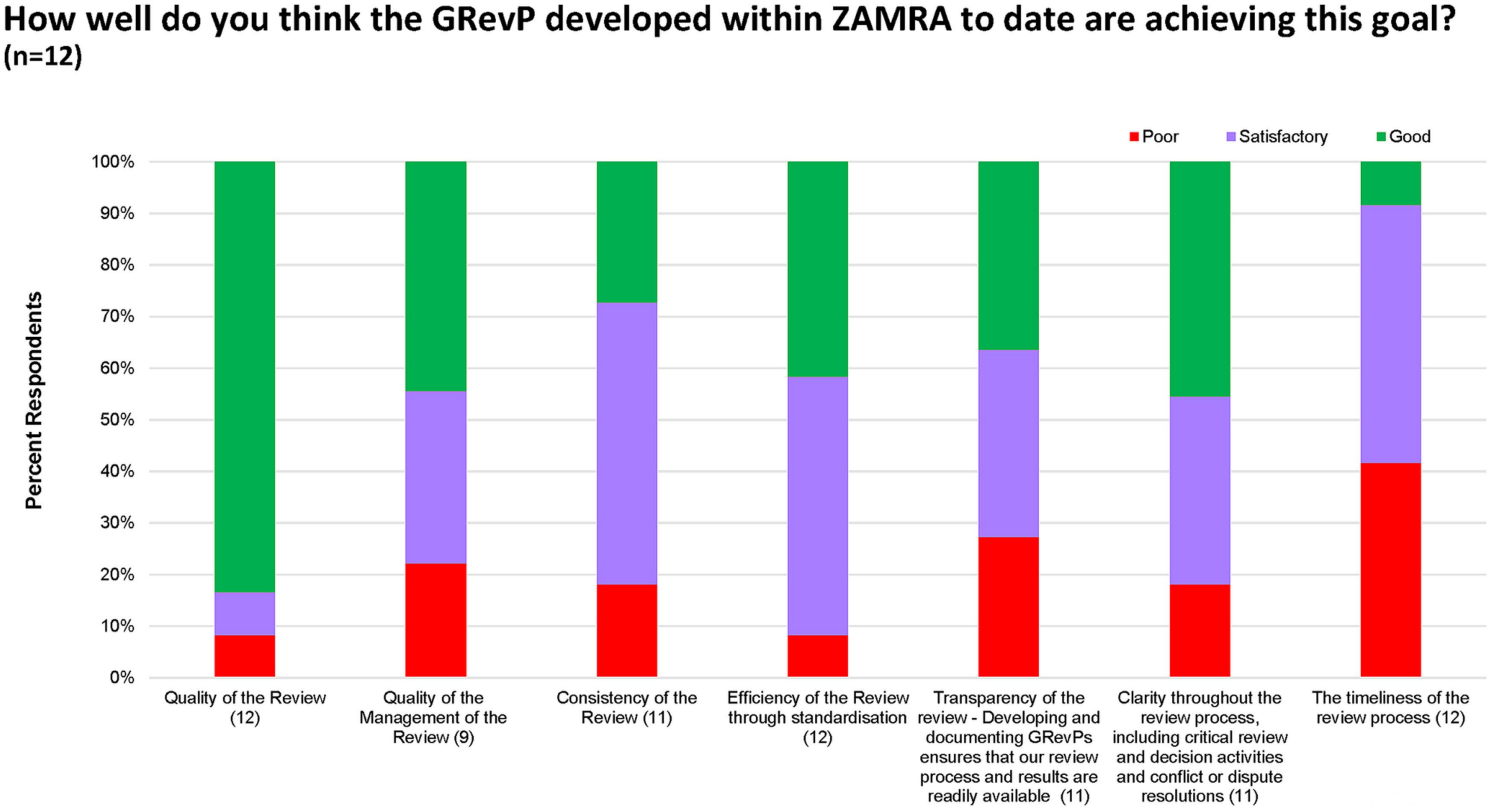
Rating on the development of GRevP within ZAMRA.

When asked whether the GRevPs developed within ZAMRA were achieving their intended purpose, study participants provided insights concerning the quality, consistency, transparency and timeliness of reviews. Concerning the quality of the review, 10 (84%) participants rated this as good and 1 (8%) satisfactory, or 1 (8%) poor. Management quality was considered good by 5 (44%), satisfactory by 4 (34%), and poor by 3 (22%) of participants. Regarding consistency, 7 (54%) respondents rated it as satisfactory, 3 (28%) respondents rated as good, and 2 (18%) respondents rated as poor, indicating the need for improvement. Efficiency through standardisation was a noted concern with half of the respondents (50%) stating that this required improvement, 5 (42%) respondents rated it as good, and 1 (8%) as poor. Transparency was seen as good by 4 (36%) respondents, satisfactory by another 4 (36%) respondents, and poor by 3 (28%), highlighting room for greater openness in the process. Clarity of the review process was viewed positively by 9 (82%) respondents, while 2 (18%) indicated it was lacking. In contrast, timeliness emerged as a key weakness with only 5 (41%) of study participants indicating this practice as good, 4 (32%) as satisfactory, and 2 (18%) as poor (Figure 4).

**Figure 5:**
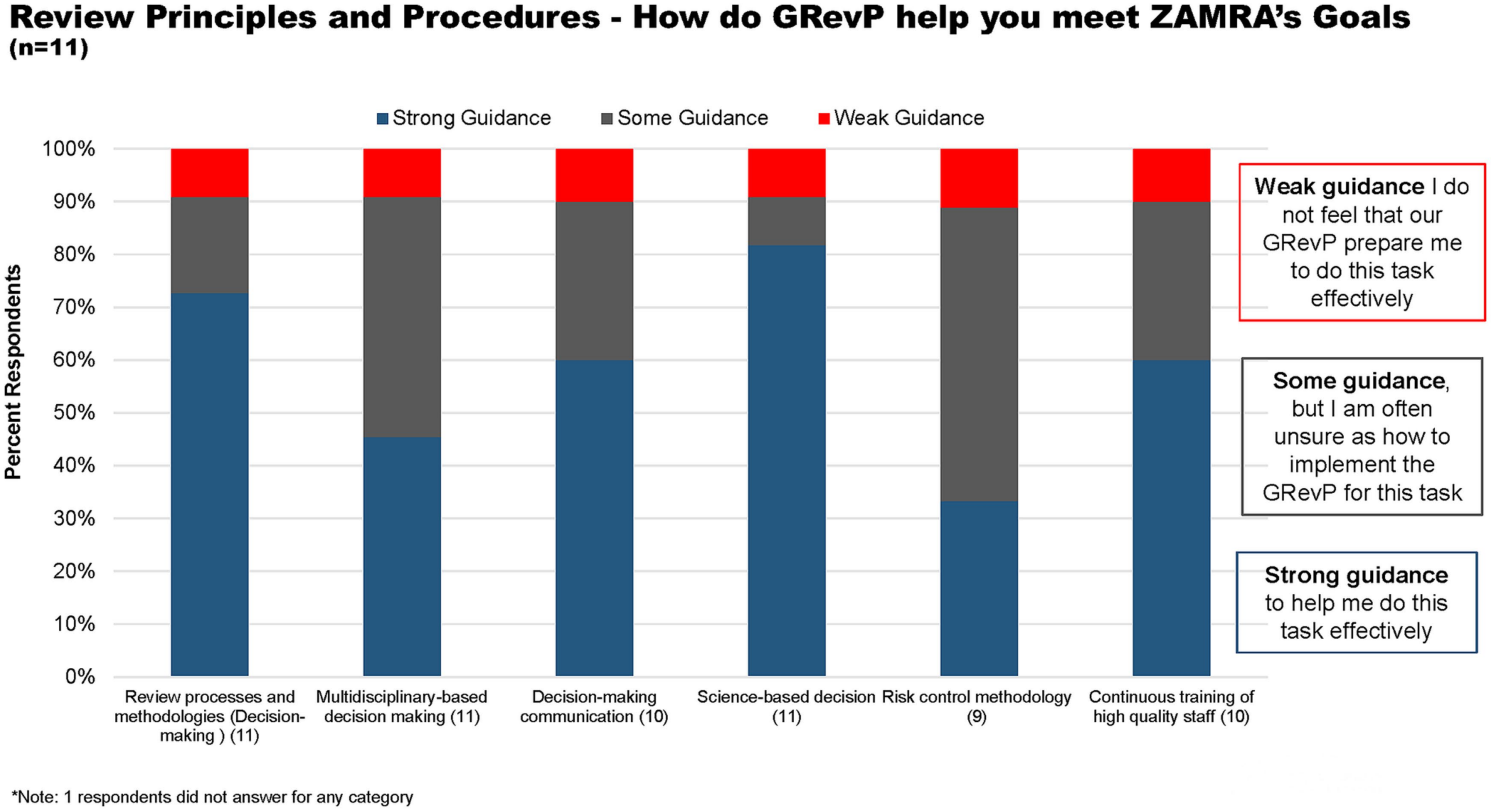
How does GRevP help meet ZAMRA’s goals through review principles and procedures.

With regard to review principles and procedures, over 72 and 82% of respondents believed that ZAMRA gives strong guidance to help them do their task through review processes and methodologies, and science-based decisions, respectively (Figure 5). Furthermore, 42 and 55% believed that the Authority gives some guidance through multidisciplinary-based decision-making and risk-control methodology but are unsure as to how to implement the GRevP (Figure 5).

**Figure 6:**
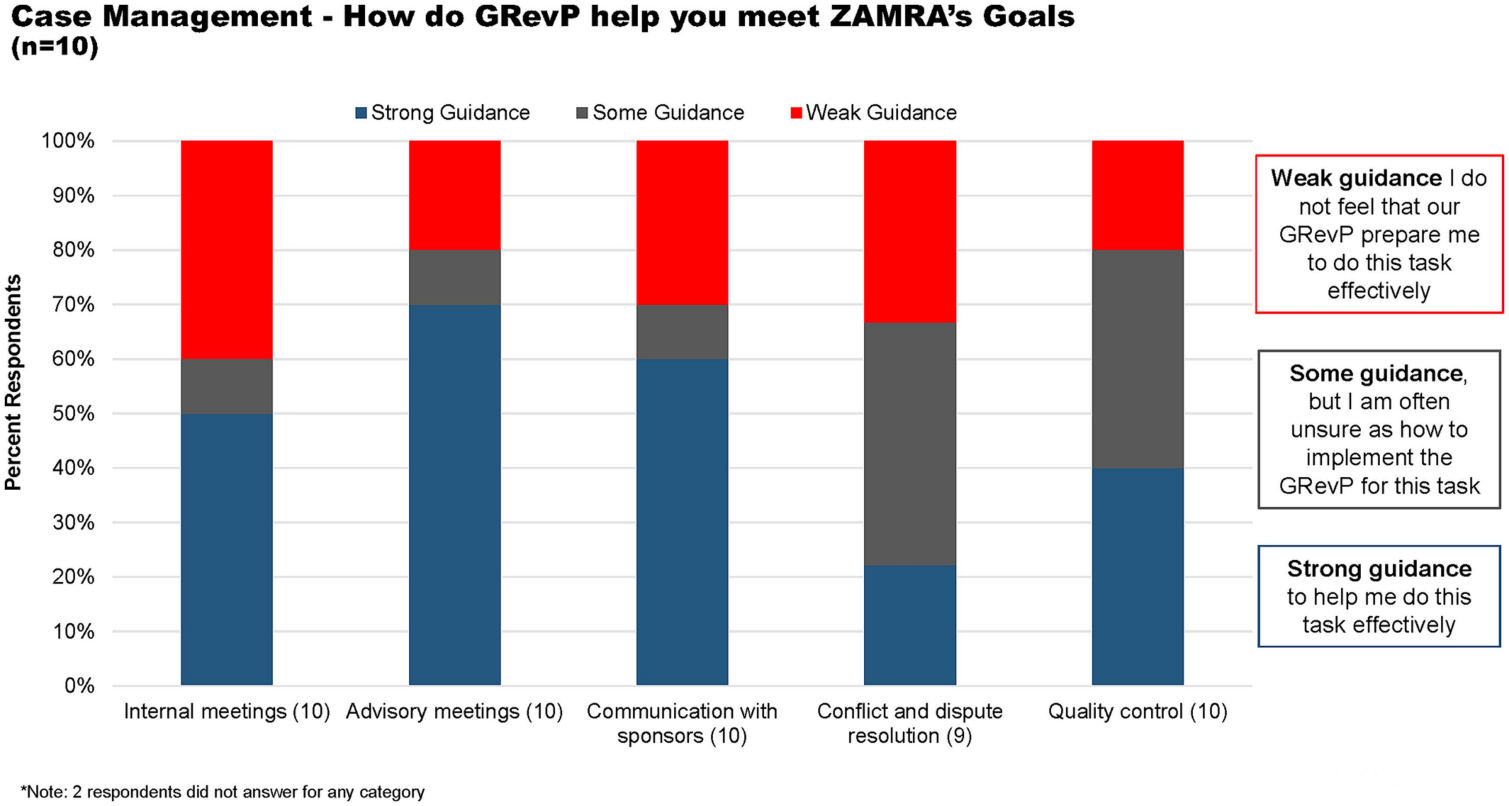
How does GRevP help meet ZAMRA’s goal through case management.

Concerning case management, 50, 70, and 60% of the respondents felt that there is strong guidance available to assist them in conducting internal meetings, advisory meetings, and communication with sponsors, respectively. Meanwhile, 40% of the respondents believed that some guidance was provided for resolving conflicts and disputes, as well as for quality control (Figure 6).

When asked what main mechanisms are employed to ensure that GRevPs are adopted, 59% of the respondents indicated that mentoring by supervisors and 41% indicated through training and follow-up by training teams or people assigned to have these implemented. The approach used by the Authority and ZAMRA is that, as GRevPs are being developed they are made available to review staff to adopt into their daily review activities. A total of 9 (75%) of the respondents indicated that they are not given formal testing on their understanding of what GRevPs are and how they should be used, 2 (17%) respondents indicated that they are given formal testing while 1 (8%) indicated that they are still in draft.

When asked what could be done better to improve the way GRevPs are implemented by ZAMRA, study participants suggested the following tasks at an individual level:

- Map processes in the GRevP of ZAMRA that align or depend on my primary responsibilities. Develop processes, QP, and templates that answer to the purpose/goals of GRevPs for collective review.
- Participate more in the implementation process.
- Training of junior staff.
- Take it upon myself to go through the guidelines and abide by them.
- Read more about GRevP. I could use reviews to improve.
- Adoption of GRevP in review.
- Continuous reference to the available guidelines and in-process standards, and use of the approved tools in my practice of duty.
- Actively participate in implementation of the same.
- GRevPs can be improved by continuous training for all assessors.
- Adhere to the principles of GRevP.

When asked what they thought the senior managers could do to improve the way GRevPs are implemented, the following were suggested:

- Collective review of proposed processes for transparency. Create clarity on the purpose of work processes. Timeliness of developing processes—proactive to purpose and not reactive pressure to record achievements, though good.
- Enhanced communication with junior staff when GRevPs are implemented.
- Providing more training from experts, e.g., Exchange programmes, External tutors, etc. Ensuring clear SOPS are available.
- Communicate effectively on the guidelines in place. Ensure the guidelines are circulated to all the assessors, emphasising their use.
- There is a need for them to emphasise the requirement for GRevP. Use reviews to drive continuous improvement. They could encourage constructive feedback. They could consider incentives.
- Develop GRevP tools and mechanisms > train staff on GRevP > assess and review implementation of GRevP by staff > improve through re-training and mentorship
- Speed up the process/finalisation of the document
- Enhance proper communication to staff: Prioritise capacity building and training of staff
- Senior managers can track the progress of individual assessors to ensure the team is compliant to GRevP
- Develop a QA team for assessed reports: Conduct refresher training in assessments and GRevP

According to 5 (42%) of the participants the statement which best represents how GRevPs are maintained/improved within the department and within ZAMRA in general is to develop the GRevP, implement, monitor and train, evaluate, improve and continue this cycle on an ongoing basis and once they have been developed GRevP are reviewed on an as needed basis.

**Figure 7:**
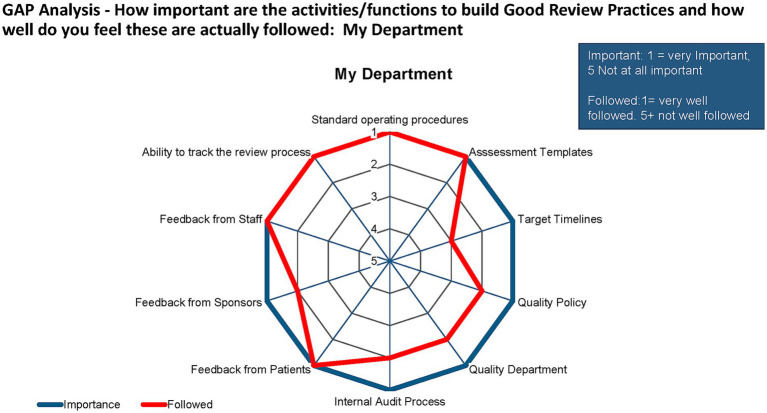
Gap analysis of the department.

A gap analysis of the importance of GRevPs for the department/individual and how closely these were followed up showed that the study participants perceived that all aspects of GRevPs were important. However, the internal audit process, quality department, quality policy, target timelines, assessment templates, feedback from patients, and staff were considered to be very important (Figure 7).

**Figure 8:**
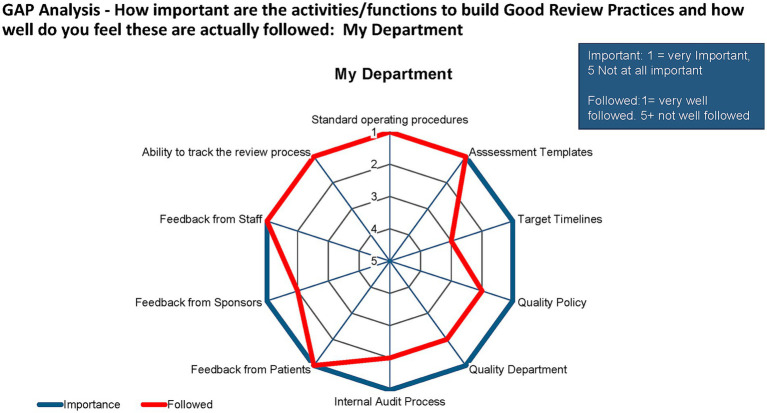
Gap analysis of ZAMRA.

A gap analysis of the importance of GRevPs for ZAMRA and how closely these were followed up showed that the study participants perceived that all aspects of GRevPs were important. However, assessment templates, target timelines, quality policy, quality department, internal audit process, feedback from patients, sponsors, and staff, and the ability to track the review process were considered to be very important (Figure 8). It was noted that the practices are mostly in parallel with perception for most aspects of GRevPs, it was however noted that with regard to target timelines, the median values showed differences between perception and practice.

## 4 Discussion

This study assessed the strategies that are implemented to develop and maintain the quality of the review processes in the Marketing Authorisation Department within ZAMRA. The results provided a valuable insight into the perception of the reviewers within ZAMRA. This study formed a baseline regarding the current knowledge, practice, and attitude within the Authority and provided an understanding of the current quality processes and areas of the review process that need continuous improvement. While some participants demonstrated limited theoretical knowledge of GRevPs, they nevertheless exhibited good practical adherence to procedural elements, suggesting experiential learning and partial implementation. It is clear that ZAMRA needs to improve training in GRevPs to ensure that all reviewers have good knowledge of these practices.

The Authority has adopted the use of standard operating procedures (SOPs), and assessment templates, which form the building block of good review practices (14). It is clear from the results obtained from this study that routine monitoring of these strategies should be enhanced to improve the quality of the regulatory review process. Though the assessors utilise some of the elements of GRevPs in their review, they do not fully always understand the principles of GRevPs; therefore, indicating a need for ongoing training. Sithole et al. (15) assessed the implementation of good review practices used within ZAMRA and the results indicated that all the eight including having review practice system, internal quality policy, peer review system, dedicated quality department, scientific committee, shared and joint reviews and standard operating procedures for guidance of assessors and use of assessment templates were implemented at the time, the only gap was with transparency and communication as the public assessment report for approved products were not published.

This study has identified several areas that require improvement within the regulatory review process, however, it also acknowledges existing strengths, such as the relevance of patient feedback in regulatory decision-making. Patient feedback is well noted as an important element in the regulatory decision-making process as patients provide unique insights into their diseases and treatment (16, 17). While not currently noted as a gap for ZAMRA, the integration of patient feedback could be further enhanced to align with global best practices. The WHO listed Authorities, such as Health Canada, EMA and USFDA have included patient feedback into benefit–risk assessments and the regulatory decision-making process throughout the product lifecycle, demonstrating that they recognise the value of the patients’ perspectives in the process (17). The United States’ USFDA and EMA have incorporated patient associations in the decision-making process, allowing them to provide their perspectives on the medicines to be approved (18).

Owusu-Asante et al. (19) conducted the study to assess the knowledge, practice and attitude of GRevPS by the Ghana FDA reviewers and it was observed that they had implemented and fully adopted the GRevPs across the agency. Though they still believed that the process could be improved. The results indicate that Ghana FDA conducts good training and has embedded GRevPs into the culture of the agency. ZAMRA on the other hand needs to embed GRevPs into the culture of the agency. Unlike the ZAMRA, Ghana FDA adheres to set timelines even though it does not have an electronic system to track their applications. Both agencies do not publish the public assessment reports and can both improve in this area to enhance transparency and communication.

The Authority has a service charter that outlines the target timelines based on the type of application (14, 20). Adherence to the set target timelines is inadequate due to lack of human resource therefore impacting the timely availability of medicines to the patients. Studies have demonstrated that adherence to set target timelines coupled with a quality regulatory review process ensures availability of quality, safe and efficacious medicines for patients (21, 22). The Authority needs to adhere to the set target timelines to ensure the availability of medicines to the general public. With the introduction of an Integrated Regulatory Information Management System (IRIMS), the Authority is now able to track its applications which are submitted electronically (23).

The consistency of assessments was perceived as variable, with feedback from respondents highlighting the need for a more structured and standardised review processes. Consistency is closely linked to the implementation of structured frameworks for quality decision-making, which promotes objectivity, reproducibility, and fairness in assessments (24). Findings by Bujar et al. (25) demonstrated the applicability of tools like the Quality of Decision-Making Orientation Scheme (QoDoS) in identifying both favourable and unfavourable practices, and in assessing the consistency and transparency of quality decision-making practices (QDMPs) within and across organisations. A well-established quality management system (QMS) is central to supporting these practices and although ZAMRA is committed to continuous quality improvement, the current lack of structured training in decision-making limits internal consistency of assessments (14).

An additional area that the study identified as needing improvement is transparency. Publication of the summary of evaluation reports for applications assessed improves transparency as applicants and other stakeholders are able to see and reflect on the processes undertaken to reach a decision. Literature shows that NRAs with advanced regulatory systems, such as Australia’s Therapeutic Goods Administration (TGA), publish their assessment reports (26). Very few NRAs in Africa publish their assessment reports, with Tanzania Medicines and Medical Devices Authority (27) the first NRA to implement such a practice. A well-established quality management system ensures that final decisions on an assessment are based on the balance between benefits and harms. The benefit–risk assessment is then communicated to the applicant, patients and the healthcare professionals through public assessment reports (28). Currently, ZAMRA does not publish the summary of evaluation reports, and it is therefore recommended that the Authority aims to emulate mature agencies and publish the assessment reports to improve transparency practices.

In view of this the Authority should publish product information, i.e., the summary of product characteristics (SmPC) as one of the requirements for WHO ML3 status which the Authority is striving to attain. There is a pressing need to strengthen regulatory review systems in emerging market economies as highlighted by the World Health Organisation (WHO) through improved transparency, having a better understanding of GRevPs and the need to be embedded in the culture of the agencies’ assessors through training. These diverse challenges may seem overwhelming to individual national regulators, in part because of the sheer number of initiatives by multiple stakeholders, combined with a lack of information on concise practical actionable measures that can have a positive impact on review efficiency’ (9). It is hoped that ZAMRA will take the necessary steps to address the gaps that have been identified in this study in order to have an improved regulatory review system and ultimately achieving WHO maturity level 3 status.

## 5 Recommendations

The following recommendations are made from the study:

- To improve the knowledge base on GRevPs for reviewers through in-house training and orientations.
- To enhance the use of the good review practices in the review process of medicines.
- To enhance timeline monitoring through the IRIMS in line with the service charter.
- To finalise and publish the ZAMRA Good Review Practices guidelines.
- Enhance transparency and communication through publishing product information including summary of product characteristics (SmPC) and patient information leaflet (PIL) for approved medicines.

## 6 Conclusion

This study assessed the level of knowledge, practice, and attitude that reviewers have and the implementation of Good Review Practices within ZAMRA. It was observed that the Authority was implementing some elements of GRevPs, while other areas, such as transparency, and communication, as well as conducting on-the-job training and mentoring the reviewers on the principles of GRevPs require improvement. Implementation of all the elements of the GRevPs will significantly improve the quality of the review process, thereby ensuring timely accessibility of quality medicines for patients. This study forms the starting point for future assessment and continuous improvement of the review process by ZAMRA, an important practice of a well-functioning regulatory system as it strives to attain WHO ML3 status.

## Data Availability

The original contributions presented in the study are included in the article/supplementary material, further inquiries can be directed to the corresponding author.
